# TITANIUM CLIPS FOR CLOSURE OF THE PERITONEAL FLAP DURING LAPAROSCOPIC INGUINAL HERNIA REPAIR

**DOI:** 10.1590/0102-672020220002e1676

**Published:** 2022-09-09

**Authors:** Eduardo Neubarth Trindade, Eduardo Ferreira Martins, Manoel Roberto Maciel Trindade

**Affiliations:** 1 Porto Alegre University Hospital, Digestive System Surgical Unit – Porto Alegre (RS), Brazil.

**Keywords:** Hernia, Hernia, Inguinal, Laparoscopy, Titanium, Surgical Instruments, Hérnia, Hérnia Inguinal, Laparoscopia, Titânio, Instrumentos Cirúrgicos

## Abstract

**BACKGROUND:**

The laparoscopic approach for inguinal hernia repair has been widely used since its introduction in the 1990s. As a step in the procedure, the surgeon must access the preperitoneal space through an incision in the peritoneum, creating an adequate dissection for mesh placement. At the end of the procedure, the peritoneal flap must be closed to avoid adhesions. There are several methods to close the peritoneum.

**AIMS:**

The aim of this study was to propose a simple method for closing the peritoneal flap, using titanium clips, exposing its advantages and disadvantages.

**METHODS:**

Description of the peritoneum flap closure technique, using titanium clips, in the last 15 years.

**RESULTS:**

The pneumoperitoneum was reduced to a pressure of 7 mmHg; then, the two edges of the peritoneal flap were approximated together and, with the aid of a Maryland grasper, were kept together; titanium clips were used to close the flap. The process is repeated along the entire peritoneal incision until it is completely closed.

**CONCLUSIONS:**

The use of titanium clips proved to be a fast, inexpensive, and effective method for closing the peritoneal flap in videolaparoscopic inguinal hernioplasties, with no major or recurrent complications reported. Therefore, it is an effective and safe method for the closure of the peritoneal defect.

## RESUMO

**RACIONAL:**

A via de acesso laparoscópica para correção de hérnia inguinal tem sido amplamente empregada desde sua introdução na década de 1990. Como etapa do procedimento, o cirurgião deve acessar o espaço pré-peritoneal por meio de uma incisão no peritônio, criando uma adequada dissecção para a colocação da tela. Ao final do procedimento, o retalho peritoneal deve ser fechado para evitar aderências. Existem vários métodos para fechar o peritônio.

**OBJETIVOS:**

Propor um método simples para o fechamento do flap peritoneal, utilizando clipes de titânio, expondo suas vantagens e desvantagens.

**METHODS:**

Descrição da técnica de fechamento do peritônio, utilizando clips de titânio, nos últimos 15 anos.

**RESULTADOS:**

O pneumoperitôneo foi reduzido até uma pressão de 7mmHg; em seguida, as duas bordas do retalho peritoneal foram aproximadas e, com auxílio de uma pinça Maryland, foram mantidas unidas; o clipe de titânio foi utilizado para o fechamento do retalho. A técnica foi repetida ao longo de toda a incisão peritoneal, até o seu fechamento completo.

**CONCLUSÕES:**

O uso de clipes de titânio mostrou-se um método rápido, barato e eficaz no fechamento do flap peritoneal nas hernioplastias inguinais videolaparoscópicas, sem grandes ou recorrentes complicações relatadas. Portanto de um método eficaz e seguro para o fechamento do defeito peritoneal.

## INTRODUCTION

The laparoscopic approach for inguinal hernia repair was first described in the early 1990s^1^. Since then, it has become very popular^10^ because of the benefits when compared to the open procedure, like the quicker return to work and less chronic pain.

The transabdominal preperitoneal (TAPP) technique is one of the most performed procedures in laparoscopic inguinal hernia repair^10^. It involves the opening of the peritoneum to access the preperitoneal space for an adequate dissection of the Fruchaud myopectineal orifice and a correct positioning of the mesh^3^.

There is still a debate on which technique is better, TAPP or totally extraperitoneal repair^11^, regarding the advantages and disadvantages of each one. One of the most recognized disadvantages of the TAPP technique is the potential risk of intestinal obstruction caused by adhesions between the mesh and bowel^2^. To avoid this complication, the peritoneal flap and peritoneal tears must be closed after the placement of the mesh.

Various methods of peritoneal flap closure have been described: suture, staples, and hemolok clips^9^. There is no consensus on which one is the best — each method has different advantages and disadvantages.

The objective of this study was to report our experience in closing the peritoneum with titanium clips.

## METHODS

Description of the peritoneum closure technique, using titanium clips, in the last 15 years.

## TECHNIQUE

**Figure 1:**
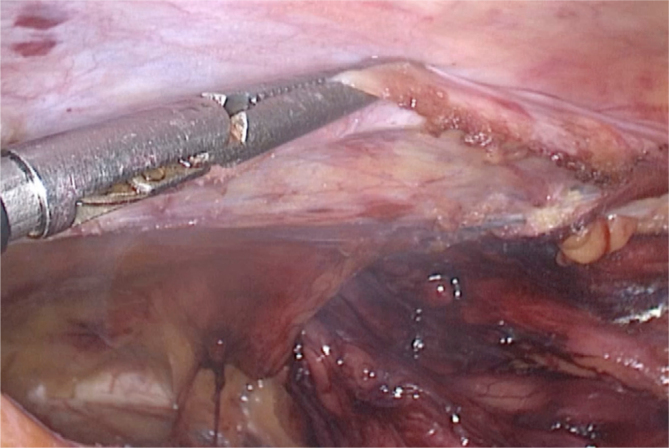
Detaching the cranial part of the peritoneal flap.

The first step is to detach the upper part of the peritoneal flap from the abdominal wall. We commonly use a Maryland grasper to pinch the peritoneum and moving it posteriorly and cranially along the entire peritoneal incision (Figure 1). In this way, we create a suitable space for the allocation of titanium clips. At this point, it is important to reduce the pneumoperitoneum to a maximum pressure of 7 mmHg in order to facilitate the approximation of the peritoneal edges.

**Figure 2:**
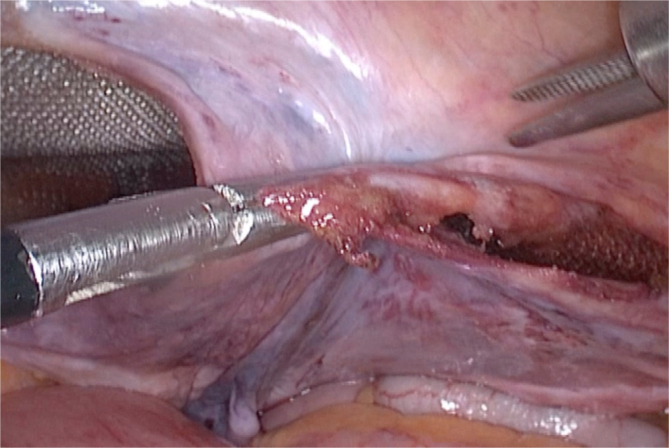
Holding the two edges of peritoneal flap with the Maryland grasper.

**Figure 3:**
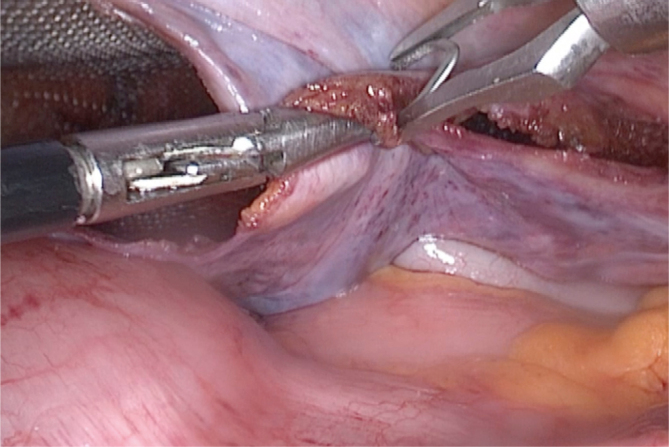
Application of titanium clip.

Afterward, we approximate the two peritoneal edges. With the left hand, we hold them together with a Maryland grasper or a straight dissecting forceps (Figure 2). Then, we use an LT300 titanium clip to definitely close the peritoneal tear (Figure 3).

**Figure 4:**
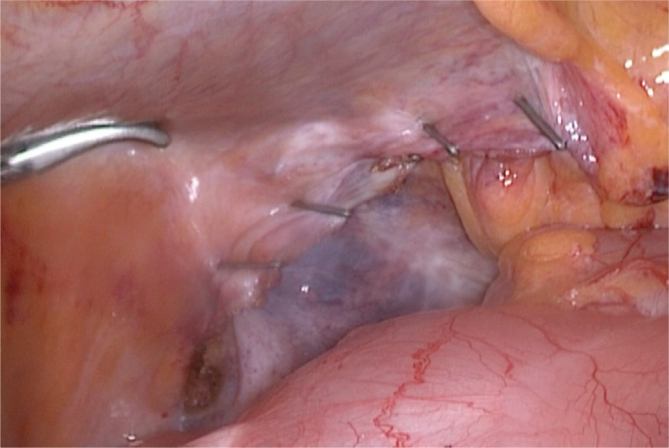
Closing the entire peritoneal flap with titanium clips.

Using the clip applicator, we bring the distal portion of the peritoneal flap closer to the proximal portion, which is more fixed to the abdominal wall. We join the two portions together with the left hand again, still using a Maryland or a straight dissection forceps. After that, we repeat the process until the entire peritoneal incision is closed (Figure 4).

We usually respect a distance of about 1–1.5 cm between clips (this distance is based on the surgeon's visual measurement). Generally, we employ 5–7 titanium clips to close the entire peritoneal flap; this amount can vary, depending on the size of the incision and the surgeon's judgment.

Eventual peritoneal tears in the distal flap can also be corrected with titanium clips, using the same technique described above.

## RESULTS

Our group has been using this technique for closing peritoneal tears for 15 years. About 600 procedures have been performed all over these years using this method. There is no report of complications, such as intestinal obstruction due to incarceration of small bowel into the preperitoneal space.

## DISCUSSION

Minimally invasive procedures are increasingly becoming the standard choice for inguinal hernia repair, since they were introduced in the 1990s — TAPP is one of the most employed techniques.

Closing the peritoneum at the end of the procedure is an important step to prevent one of the most feared complications in the postoperative period: intestinal obstruction due to herniation into the preperitoneal space^2^. It is not a common condition, with an estimated incidence of about 0.28%^4^, but it can be life-threatening.

Some authors advocate that peritoneal closure must be performed by a technique that promotes a complete and continuous approximation between the two peritoneal edges. Using this definition, suture would be the most adequate technique^8^. However, it has been reported that cases of small bowel obstruction were related to self-anchoring suture used for peritoneal closure after laparoscopic inguinal hernioplasty^5,6^. Furthermore, there are other disadvantages of suturing the peritoneal defect: increased operative time, technical difficulty, and inadequacy for very thin peritoneum^7^.

Titanium clips are made of an inert material that is widely used in other procedures such as cholecystectomy. It can be an alternative to suturing the closure of the peritoneum flap, with some advantages and disadvantages. This technique can be easier and faster to perform compared to suturing, contributing to the reduction of operative time.

However, some disadvantages of this technique were noted. One of the most notable is the risk of intestinal obstruction due to herniation of a portion of the small intestine into the preperitoneal space through the gaps between the clips. To avoid this, the clips must be applied at a maximum distance from each other — we normally respect a distance of about 1–1.5 cm; eventually, the gaps between the clips must also be closed, depending on the surgeon's judgment.

As a step of the procedure, the cranial edge of the flap must be detached from the abdominal wall to create a redundancy of the peritoneum for a suitable installation of a titanium clip. Another possible disadvantage is the difficulty to pinch the superior part of the peritoneal flap. In these cases, there is no redundant peritoneum, which aggregates technical obstacles to the procedure.

## CONCLUSIONS

There are various methods to close the peritoneal flap described; all of them have their advantages and disadvantages. Using titanium clips to close the peritoneum is an efficient method, easy to apply in most cases, and faster than other methods, such as suture. The most feared complication is the intestinal obstruction due to herniation of small bowel into the preperitoneal space through a gap between the clips; it can be avoided or, at least minimized, if a suitable distance clip-to-clip be respected.
